# Luminescence in Sulfides: A Rich History and a Bright Future

**DOI:** 10.3390/ma3042834

**Published:** 2010-04-21

**Authors:** Philippe F. Smet, Iwan Moreels, Zeger Hens, Dirk Poelman

**Affiliations:** 1 LumiLab, Department of Solid State Sciences, Ghent University, Krijgslaan 281-S1, Gent, Belgium; E-Mail: dirk.poelman@ugent.be (D.P.); 2 Physics and Chemistry of Nanostructures, Department of Physical and Inorganic Chemistry, Ghent University, Krijgslaan 281-S3, Gent, Belgium; E-Mails: iwan.moreels@ugent.be (I.M.); zeger.hens@ugent.be (Z.H.)

**Keywords:** sulfides, photoluminescence, electroluminescence, phosphor, rare earth, nanocrystals, quantum dots, europium, cerium, light emitting diodes, persistent luminescence, storage phosphor

## Abstract

Sulfide-based luminescent materials have attracted a lot of attention for a wide range of photo-, cathodo- and electroluminescent applications. Upon doping with Ce^3+^ and Eu^2+^, the luminescence can be varied over the entire visible region by appropriately choosing the composition of the sulfide host. Main application areas are flat panel displays based on thin film electroluminescence, field emission displays and ZnS-based powder electroluminescence for backlights. For these applications, special attention is given to BaAl_2_S_4_:Eu, ZnS:Mn and ZnS:Cu. Recently, sulfide materials have regained interest due to their ability (in contrast to oxide materials) to provide a broad band, Eu^2+^-based red emission for use as a color conversion material in white-light emitting diodes (LEDs). The potential application of rare-earth doped binary alkaline-earth sulfides, like CaS and SrS, thiogallates, thioaluminates and thiosilicates as conversion phosphors is discussed. Finally, this review concludes with the size-dependent luminescence in intrinsic colloidal quantum dots like PbS and CdS, and with the luminescence in doped nanoparticles.

In this Review, we discuss the rich and longstanding history of sulfide phosphor materials, dating back from at least the 17th century. Progress in the understanding of the basic principles of luminescence culminated in several typical applications, uniquely based on sulfides, such as ZnS-based powder electroluminescence and thin film electroluminescence. Turning towards the 21st century, sulfide-based nanoparticles and color conversion phosphors possess several characteristic properties which give them a bright future as well.

The paper is structured as follows:

1. Sulfide phosphors: A short history
2. Electroluminescent powders
3. Lamp and CRT phosphors
4. Thin film electroluminescence
5. Color conversion phosphors
6. Persistent and storage phosphors
7. Luminescent sulfide nanoparticles
8. Conclusions

## 1. Sulfide phosphors: A Short History

Luminescent phenomena have fascinated mankind since the earliest times. The light from the aurora borealis, glow worms, luminescent wood, rotting fish and meat are all examples of naturally occurring luminescence. The effect was shrouded in mystery, and described accordingly in the Middle Ages and beyond. The earliest written account of a solid state luminescent material comes from a Chinese text published in the Song dynasty (960–1279 A.D.), referring to a book (never recovered) from the period 140-88 B.C. It describes a painting of a cow grazing outside. In the dark, the cow would have been seen resting inside a barn [1,2]. Possibly, the ink used was the first man-made persistent phosphor material. Harvey [3] presents an excellent account of these early observations far beyond the scope of the present review.

The first artificial phosphor described in Western literature dates from 1603. Then, the Italian shoemaker and alchemist Vincenzo Cascariolo used the natural mineral barite (BaSO_4_), found near Bologna, in an effort to create gold. After heating the ground stone under reducing condition he–obviously – did not obtain gold, but a persistent luminescent material. This so-called Bolognian stone became famous and a subject of study and admiration for decades to come [3]. It is not clear which dopant or dopants were actually responsible for the persistent luminescence, but the host material [2] definitely was BaS. While not made intentionally but by serendipity, BaS thus is the first sulfide phosphor ever synthesized. The name phosphor (from the Greek ‘light bearer’) was already used at that time, even if the chemical element phosphorous was only isolated in 1669 (from urine) by the German alchemist Hennig Brand [1]. Phosphorous becomes luminescent under moist conditions, when it oxidizes. Thus, phosphorous is chemiluminescent and the name phosphorescence for persistent ‘glow in the dark’ photoluminescence is actually a bit of a misnomer [4].

In the following centuries, many scientists synthesized and investigated luminescent materials, but it was too early for a systematic study. However, the synthesis of CaS as a phosphor in 1700 by Friedrich Hoffmann and of SrS in 1817 by J. F. John are worth mentioning. Curiously enough, the luminescent properties of ZnS, which was going to become one of the most important luminescent hosts in the 20th century, were not recognized until 1866, when the so-called Sidot blend (hexagonal ZnS) was developed by Theodor Sidot [5]. In 1888, Eilhard Wiedemann was the first to classify different classes of phosphors according to the type of excitation, and is credited for introducing the terms luminescence, photoluminescence, electroluminescence, thermoluminescence, crystalloluminescence, triboluminescence and chemiluminescence [6].

## 2. Electroluminescent Powders

Already in 1907, H. J. Round published light emission from a silicon carbide junction diode, the first light emitting diode (LED) ever. Independently, Losev observed emission from ZnO and SiC diodes, as published in 1927 [7]. However, as LED’s are injection electroluminescent devices and contain no phosphors, we will not deal with this kind of devices further on.

Destriau is credited for the discovery of phosphor-based high field electroluminescence in solids in 1936 [8]. The original Destriau cell consisted of a Cu-doped ZnS powder in castor oil, insulated from one of the electrodes by a mica sheet. The applied AC voltage was very high and the light emission very poor, leading to the suspicion that the actual light emission was not due to electroluminescence by excitation of the ZnS:Cu, but due to the photoluminescence of the ZnS:Cu, excited by the UV emission of electrical discharges in gases in the porous powders [4,9]. In the following years, planar electroluminescent devices were developed, helped by the availability of SnO_2_ as a transparent conductor. EL panels were incorporated for dashboard back illumination from the late 1950’s, for example in the Chrysler Imperial 1960 luxury car. In an effort to reduce the size and energy consumption of displays, 7 segment electroluminescent numerical displays were used in the Apollo program DSKY (display panel and keyboard) module instead of the traditional nixie tubes. Quite luckily for the developers of EL devices, the repeated failures of a segment of this display during the Apollo 11 mission were later attributed to a faulty driving circuit [10] and not to problems with the display itself.

Many research groups were active in the research on powder EL, but especially the contributions by Thornton [11], Piper and Williams [12], and Vecht [13] should be noted. The research on powder EL has been marked by periods of intense research and success followed by periods of disillusion and discouragement. At the beginning and the middle of the 1960s, a series of books and book chapters gathered the – now largely forgotten – knowledge accumulated during the former phase [14,15,16,17,18,19,20,21].

AC powder electroluminescent devices (ACPEL devices in short) typically consist of a doped ZnS powder suspended in a dielectric binder, sandwiched between electrodes and supported on a substrate. The substrate can be metallic or insulating (glass or plastic). An additional white reflecting layer could provide additional electric protection and improved light output from the device.

Similar DCPEL devices require a highly conductive surface layer for current injection into the phosphor particles. Devices are prepared using Cu concentrations higher than the solubility limit in ZnS. While the surface excess Cu is washed away in the case of ACPEL devices, it is converted into an inhomogeneous conductive layer using an electrically-assisted forming process. Several models have been proposed on the exact mechanism of this process, but there is evidence of the formation of needle-like Cu_2-x_S phases. As Cu_2-x_S is p-type and ZnS is weakly n-type, this could lead to an improved carrier injection in the ZnS particles. In addition, DCPEL phosphors require a very monodisperse and small particle size in order to limit current inhomogeneity and electric breakdown. Copper is thus essential in all DCPEL devices, acting both for current injection and as a light emitting dopant. Next to copper, manganese has been used extensively as a dopant in both AC and DC powder EL devices, improving brightness and increasing the possible color gamut.

DCPEL panels are – in principle – ideally suited for graphical displays. A few commercial applications have emerged, which are now superseded by other display technologies. There is very little recent research interest in DCEL. A detailed review on DCEL devices was written by Chadha [9].

AC powder electroluminescent devices are still used in the niche application of very thin, low light level, low cost, large area background lights on flexible substrates, such as electronic gadgets, cell phones, remote controls and car radios. A number of issues prevent their widespread use:

- The absolute brightness is quite low. As large areas can emit quite homogeneously, the total light output can be considerable, but making a sunlight readable device, requiring high surface brightness, is a problem.
- The lifetime of moderate to high luminance devices is limited. The brightness of an ACPEL device can be increased by increasing the applied voltage, but this in turn decreases the lifetime. Thus a low luminance device can last for many 1000s of hours, but this lifetime decreases drastically at increased luminance. With improvements in technology, a lifetime of about 2500 h (at 50% relative luminance) with an initial luminance of 100 cd/m^2^ can now be achieved [22]. Probably, the degradation is related to diffusion of copper or blunting of the copper needles in the phosphor layer, but this is still a matter of debate. Chen *et al.* showed that the degradation rate increases at higher operating temperatures and almost drops to zero when operated at -67 °C, suggesting diffusion related degradation [23]. Heating of degraded devices to 200 °C leads to a partial rejuvenation [24].
- The stability, and thus the lifetime, is highly dependent on the encapsulation of the layers. As these are moisture sensitive, they should be very well shielded from the ambient. First, the layers were encapsulated as a whole, but more recently, micro-encapsulation has been performed, the particles being coated individually. Obviously, this kind of additional process increases the cost of the material.
- The overall external efficiency of ACPEL devices is very low, of the order of only a few lm/W, which makes the technology unsuited for general lighting applications, and certainly not a match for CFL’s (compact fluorescent lamps) and LEDs.

As ZnS:Cu is the only material for efficient powder EL, there seems little room for drastic improvements in device performance. At best, powder EL will remain a technology for blue-green – the emission color being frequency dependent - background lighting for undemanding applications. A recent review on ACPEL can be found here [25].

## 3. Lamp and CRT Phosphors

Starting before the Second World War, many new luminescent materials were developed for fluorescent lighting. In a fluorescent lamp, the ultraviolet emission of an electrical discharge of a low pressure mercury vapor is converted to visible light by phosphor materials, covering the inside of the lamp. Sulfide phosphors are of no use in this kind of fluorescent lamps, since they react with mercury [26].

Since the advent of high performance flat panel displays a few years ago, any treatment of CRT (cathode ray tube) phosphors is – almost by definition – of historical interest. After its discovery by Braun in 1897, the CRT has had tremendous success. One of the first CRT images (the Japanese Katakana character “i”) was shown in 1926 by Takayanagi. First screens were black and white, later full color displays were taken into production, thanks to the development of a large number of highly optimized possible phosphor materials [2,26]. For blue cathodoluminescence, ZnS:Ag has been the material of preference since the beginning. It has a very efficient emission due to a donor acceptor transition: the donor level being due to an aluminum or chlorine co-dopant and the acceptor level due to silver [26]. This kind of process implies that the emission wavelength is not determined by the nature of the dopants, but by the band gap of the host. By making a solid solution of ZnS and CdS, the spectrum of Zn_1-x_Cd_x_S:Ag could be tuned from a peak wavelength of 450 to 620 nm [4]. Thus, both green and red emitting phosphors could be made using this technique. Nowadays, such Cd-containing compounds have become unacceptable for environmental reasons. For the green phosphor, ZnS:Cu (codoped with Al or Cl) is routinely used. For the red one, the line emission from Eu^3+^ was found to be an ideal compromise between optimum color coordinates and eye sensitivity [26]. The host of choice for red emission is Y_2_O_2_S. Many other cathodoluminescent phosphors were developed for specific applications, like projection displays (high excitation current), field emission displays (FED) (low voltage, high current) and flying spot equipment (fast decay times). Their study and description is outside the scope of this review. A recently compiled list of CRT and FED phosphors can be found here [27,28].

## 4. Thin Film Electroluminescence

### 4.1. Working principle

**Figure 1:**
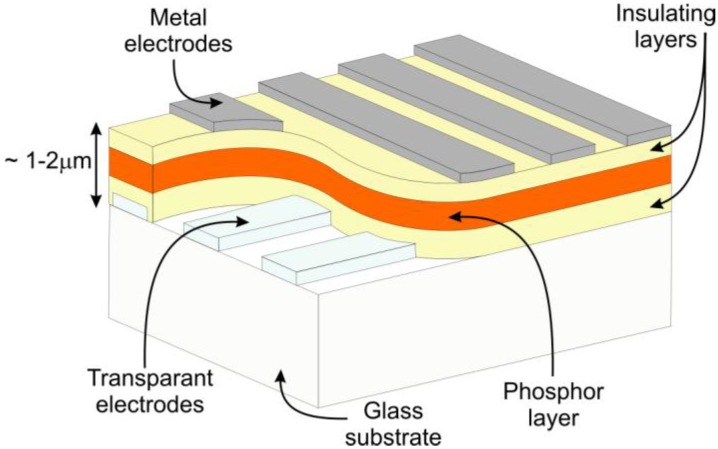
M(etal)-I(nsulator)-S(emiconductor)-I(nsulator)-M(etal) structure used for thin film electroluminescence displays (color online).

In parallel with the development of powder EL, a new type of device, using a thin film phosphor, was presented by Vlasenko and Popkov in 1960 [29]. The device used ZnS:Mn as the active layer and was much brighter than an equivalent powder EL lamp. However, stability was a problem due to the very high electric fields, needed to drive the device. This problem was largely solved by Russ and Kennedy in 1967, who proposed a double-insulated structure, protecting the active layer from destructive dielectric breakdown [30]. The resulting device structure, which is still used to date, is shown schematically in Figure 1.

When a voltage is applied over the electrodes, it is capacitively divided between the two insulators and the central active layer. As both insulating layers and active layer have a large band gap, there are a negligible number of free electrons and holes available and no current is flowing. However, there are a number of allowed energy levels at the insulator-active layer interfaces (appropriately called interface states). To a certain extent, these are filled with electrons. When the electric field is high enough, of the order of 1 – 2.10^8^ V/m, the energy bands are tilted and Fowler-Nordheim tunneling of the electrons at the cathodic interface into the conduction band of the active layer becomes possible. These electrons are then accelerated to high energies by the high electric field, and can impact/excite the activator ions in the central layer. When the activator ion returns into the ground state, light is emitted. The active layer thus acts as a ‘leaky’ capacitor in these high fields, and electrons are transported from the cathodic to the anodic insulator-active layer interface. This charge transfer creates an additional electric field opposite to the applied field, therefore the tunneling, charge transfer and light emission stop after some time, usually after some microseconds. Quasi continuous light emission is obtained by AC driving of the device: a short light pulse is emitted at each polarity switch of the applied voltage. A much more detailed discussion of the physics of these ACTFEL (AC thin film electroluminescent) devices was given by Mach and Mueller [31,32,33] and Rack and Holloway [34].

Curiously enough, it lasted until 1974 before an ACTFEL display using the device structure of Russ and Kennedy was presented [35]. In the following years, several companies started producing monochrome orange emitting displays based on ZnS:Mn, some of which are still being made. While this kind of display cannot offer the visual performance and display size of other modern flat panel display technologies, it does serve a niche market where its unique properties are needed:

- ACTFEL displays can have an unsurpassed lifetime of the order of 50.000 hours.
- As this is a fully solid state display, it can be made very rugged to withstand harsh environments, in industrial, medical, military or aviation applications.
- The tunneling mechanism, which is the cornerstone of the device operation, is essentially independent of temperature. Therefore, these displays can be made to work at extremely low and high temperatures, the temperature range of the drive electronics being the main limiting factor. An EL device has been reported to work down to 15 K [36].
- ACTFEL is an emissive display technology; therefore, the viewing angle can be very large, of the order of 170°, both horizontally and vertically.
- If transparent conductors are used for both top and bottom electrodes in Figure 1, the entire display can be made transparent [37].
- The active layer is very thin – of the order of 500 nm – therefore the display resolution can be high. A microdisplay with a pixel pitch of 24 µm was presented by Planar Systems [38].

**Figure 2:**
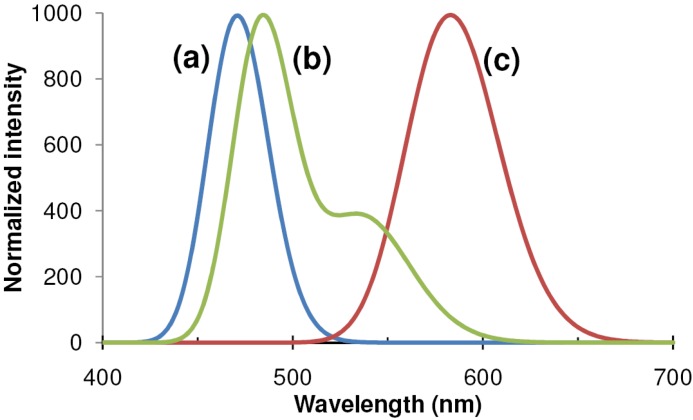
Emission spectrum of **(a)** BaAl_2_S_4_:Eu [40], **(b)** SrS:Ce,Cl and **(c)** ZnS:Mn [41].

The main drawback of ZnS:Mn ACTFEL displays was the lack of full color capability. Multicolor displays can be made by filtering the wide orange wavelength distribution of the ZnS:Mn emission to green and red [39], but RGB full color is impossible (Figure 2).

### 4.2. Towards full-color EL

In a first effort to obtain different emission colors, the Mn dopant in ZnS was replaced by trivalent rare earth luminescent centers [42,43,44], notably Tb, Er, Dy, Sm, Nd, Tm, Ho and Pr. The emission from these ions is due to well shielded 4f-4f transitions, giving rise to sharp emission peaks. As the size of the trivalent rare earth ions typically is much larger than that of the Zn cation in ZnS, it is not easy to incorporate these ions substitutionally, although higher dopant concentrations can be obtained in thin films compared to single crystals or bulk powders. For most of the rare earth ions, a rather weak emission was observed. Only ZnS:Tb (efficient green emission) [45,46], ZnS:Sm (weak red emission) [47] and ZnS:Ho (white) [48] have received some interest in later years. For ZnS:Tb, the efficiency was increased by codoping with fluorine [42] and it was shown that actually TbOF centers were formed [49], thus conserving charge neutrality.

Alternative hosts for luminescent dopants were found by returning to the well known sulfide phosphors from the 19th century. Indeed, the basic requirements for the active layer in an ACTFEL device are [50,51,52]:

- A wide band gap semiconductor is needed, as it has to be transparent to the emitted light. However, the band gap should not be too high, allowing avalanche multiplication processes.
- The dopant chosen should show an efficient emission under high electric fields, which excludes donor-acceptor based emission. Therefore ions with internal transitions are preferred, such as encountered in Mn^2+^ and the rare earth ions (both 4f-4f and 5d-4f emittors).
- The host’s cation size should match the size of the dopant ions to facilitate the substitutional incorporation in the host lattice. In addition, its oxidation state should preferably be the same as that of the dopant, although charge compensating co-dopants can be used. The ideal concentration of the dopants depends on the type of dopant, but is typically in the order of 1%. At higher concentration, non-radiative decay becomes more important because of an increased energy transfer between dopant ions. Also, high dopant concentrations can distort the host lattice thus lowering the excitation efficiency. This will especially be important for dopant ions with deviating valence state and/or ionic radius compared to the substituted ion.
- A very important parameter, which precludes the use of almost all oxides, is the need for a crystalline layer. In the applied electric field, electrons should be accelerated ballistically [53]. If the layer is amorphous, electrons are scattered at numerous grain boundaries and thus cannot gain sufficient energy to impact/excite the activator ions. While sulfides quite easily crystallize at moderate temperatures (around 500 °C), very high processing temperatures are typically needed for crystallizing oxide materials. Another effect favors the use of sulfide materials. At high electric fields (in the order of MV/cm), electron-phonon interaction is the main scattering mechanism. Hence, host compounds having low optical-phonon energies are favored. Benalloul *et al.* compared phonon energies of sulfides and oxides and observed significantly lower values for sulfides compared to oxides [54]. The optical-phonon energy for ZnS (44meV) is similar to the one in BaAl_2_S_4_ (30-40meV) [55], both being efficient EL hosts.

In the middle of the 1980s it became clear that rare earth doping of ZnS would not lead to sufficiently bright EL materials. As a result, several new activator-host combinations were tested and found to yield bright emission, CaS:Ce (green) [56], SrS:Ce (blue-green) [57], CaS:Eu (red) [58] and SrS:Eu (orange) [59] being the most successful combinations. In these phosphors, the luminescent ions are Eu^2+^ and Ce^3+^. Within the range of rare earth ions, they are exceptional in the sense that the luminescent electronic transition is due to a 5d – 4f transition, which is not well shielded from the crystal field of the host lattice. This has two effects: first of all, the emission has a broadband spectrum and secondly, the emission spectrum can be influenced by changing the host. Since several of the sulfides form solid solutions in all compositions, without any phase change, it became possible to tune the color coordinates of the emission by changing the ratio of the components in the solid solution. This fact was employed successfully in Ca_1-x_Sr_x_S:Eu (orange to red) [60,61], CaS_1-x_Se_x_:Eu (orange to red) [62,63] and SrS_1-x_Se_x_:Ce (blue to blue-green) [64,65,66]. The research on the latter two hosts was, however, abandoned due to the high toxicity of H_2_Se [67], which is liberated upon exposure of the material to moisture.

The subsequent research into improving material quality led to a prototype of a full color computer monitor type display by Planar in 1993 [68]. The way in which this display was constructed, shows the state of the art and the remaining problems at that time: The red and green pixels of the display used filtered ZnS:Mn emission, and the blue phosphor was filtered SrS:Ce. A major drawback of SrS:Ce for display applications is indeed the broad emission spectrum from Ce^3+^: the effective emission spectrum is blue-green, and the green component has to be filtered out to obtain saturated blue (Figure 2). In the prototype display in 1993, not only the size of the SrS:Ce pixels was larger than that of the ZnS:Mn pixels, but also the drive frequency of the SrS:Ce pixels was higher, both tricks meant to obtain a sufficiently intense blue emission.

In the following years, most research on ACTFEL phosphors was devoted to improving the intensity, color purity and stability of the blue component. As most sulfide phosphors are hygroscopic [69], reactions with the ambient and with the insulating layers had to be prevented. Secondly, due to the low sticking coefficient of sulfur, films prepared by PVD (physical vapor deposition) methods were sulfur deficient. This fact was usually overcome by co-evaporation of sulfur or reactive deposition in an H_2_S atmosphere. Thirdly, films deposited at low temperature by PVD processes were amorphous. In order to obtain polycrystalline layers, high substrate temperatures or post-deposition annealing treatments [70] had to be used. Finally, while the most straightforward PVD technique for sulfide films is electron beam evaporation, alternative techniques such as magnetron sputtering [71] and ALD (atomic layer deposition) [72,73] were also employed, allowing a better control of the thin film properties.

In the early 1990s, when it was realized that the (filtered) blue emission intensity in SrS:Ce remained low, research efforts were directed towards ternary sulfide hosts. The ternary thiogallates CaGa_2_S_4_ and SrGa_2_S_4_ were proposed as a new class of promising TFEL phosphors, doped with Ce or Eu [74,75]. However, these materials did not provide a real breakthrough of ACTFEL technology due to the difficulty to prepare high quality thin films that allowed sufficient electron acceleration.

In 1997, SrS:Cu and SrS:Cu,Ag were investigated for the first time as blue-emitting ACTFEL-phosphors [36,66,76,77]. In contrast to the situation of Cu as a dopant in ZnS, where a donor-acceptor transition is taking place, the emission was found to result from an internal transition of the Cu-ion. Unfortunately, the luminescence in SrS:Cu,(Ag) suffered from severe thermal quenching and dependence of the emission spectrum on the exact preparation conditions of the phosphors [66]. Indeed, in the years following 1997, several papers on the same material were published, consistently showing entirely different results.

Also in the 1990s, CaS:Pb was briefly considered as one of the best candidates for blue thin film EL [73,81,82,83,84]. Problems with clustering of the Pb ions, leading to a red shift of the emission and problems with crystallinity, prevented this phosphor becoming popular. CaS:Bi, a phosphor which had been marketed already in 1870 as Balmain’s paint, the first well-recognized commercial luminescent pigment [3], was also tested, but revealed similar problems as CaS:Pb [85].

1999 turned out to be a very important year for ACTFEL as the new blue phosphor BaAl_2_S_4_:Eu was presented by N. Miura from Meiji University, Japan [86,87], with properties that were far superior to any previously investigated material. As this is a phosphor of current interest, it will be treated in more detail in the following paragraphs. The most important thin film EL phosphors studied in the 20th century are listed in Table 1.

**Table 1:** Overview of proposed thin film electroluminescent materials with their emission color and dominant wavelength (λ_d_).

Material	Color	λ_d_ (nm)	Refs.
ZnS:Mn	Amber	585	[29]
ZnS:Tb	Green	545	[46,78,79]
ZnS:Ho	White	550	[44,48]
ZnS:Sm	Red	651	[47]
CaS:Ce	Green	505	[56]
SrS:Ce	Blue-green	480	[57]
CaS:Eu	Red	660	[58]
SrS:Eu	Orange	610	[59]
SrS_1-x_Se_x_:Ce	Blue	465	[64]
CaS_1-x_Se_x_:Eu	Orange-red	630	[62,63,80]
CaSr_1-x_S_x_:Eu	Orange-red	640	[61]
CaS:Pb	Blue	450	[73,81,82,83,84]
CaS:Bi	Blue	450	[85]
BaAl_2_S_4_:Eu	Blue	475	[86,87,88,89]
CaGa_2_S_4_:Ce	Blue	460	[90]
CaGa_2_S_4_:Eu	Yellow	565	[91]
SrGa_2_S_4_:Ce	Blue	445	[90,92,93,94]
SrGa_2_S_4_:Eu	Green	532	[71,75,94,95]
SrS:Cu	Blue-green	480	[36,77]
SrS:Cu,Ag	Blue	440	[36,76,77]
CaS:Cu,Ag	Blue	450	[96]

### 4.3. BaAl_2_S_4_:Eu and color-by-blue

The research into SrGa_2_S_4_:Ce as blue emitting phosphor was followed by the introduction of BaAl_2_S_4_:Eu as an efficient blue emitter, with a relatively narrow emission band centered around 470 nm [97]. Although briefly mentioned by Benalloul *et al.* in 1998 as a promising but difficult to synthesize material in thin film form [54], the breakthrough came in 1999 with the announcement by Miura *et al.* of ‘High-luminance blue-emitting BaAl_2_S_4_:Eu thin film electroluminescent devices’ [86].

#### 4.3.1. Deposition techniques

**Figure 3:**
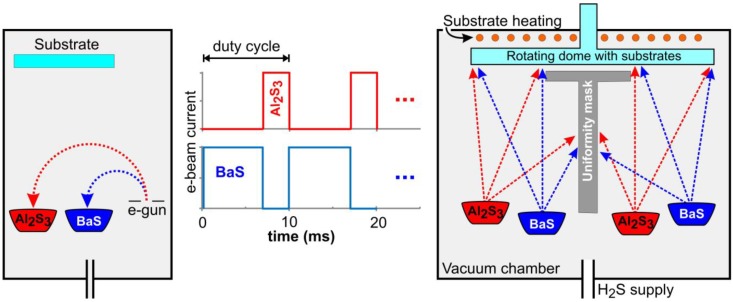
**(left)** Dual source electron beam deposition for BaAl_2_S_4_:Eu thin films, **(middle)** evaporation of both sources is obtained by rapidly switching the single electron beam (with constant current). Stoichiometry is achieved by tuning the time ratio between both sources, **(right)** multi-source modification for improved stoichiometry over large areas. (adapted from [86,104]).

Using a dual-source pulsed e-beam evaporation of BaS:Eu and Al_2_S_3_, followed by a thermal annealing at 900 °C in argon, a luminance of 65 cd/m² at 50 Hz was obtained at approximately 80 V above threshold. With CIE color coordinates of (0.12, 0.10), this phosphor was close to the requirements for the blue component in television displays. The dual-source pulsed evaporation is based on the electron beam being rapidly switched (duty cycle of 10 ms) between both sources (Figure 3), with the evaporation rate of both materials and thus the stoichiometry being determined by the electron flux ratio to both sources, which is considerably more reproducible and reliable than using thickness monitors [98].

This dual-source technique overcomes the non-stoichiometric evaporation when trying to evaporate BaAl_2_S_4_:Eu powder directly by an electron-beam. To improve the compositional and thickness homogeneity of the deposited thin films over large areas, substrates were mounted on a rotating dome with specific positioning of two BaS:Eu and two Al_2_S_3_ sources (Figure 3). In this way, five 17’’ displays could be simultaneously covered [98].

Initially, high annealing temperatures were required to obtain devices with high luminance (typically 900 °C), putting severe constraints on the substrate and the bottom electrodes and insulators. An increase of the substrate temperature from 150 °C [86] to 650 °C [99] was proposed to lower or eliminate the need for post-deposition annealing. Furthermore, a modified BaAl_2_S_4_:Eu phosphor with a partial substitution of Ba by Mg also eased the temperature requirements [100], as well as the using of fluxing agents, such as fluorides [101].

Based on research on BaAl_2_S_4_(:Eu) powders and thin films [89,102], a second crystallographic phase was identified besides the well-known, cubic phase which is obtained at high temperatures [97,103]. Upon sintering a mixture of BaS and Al_2_S_3_ powders in a flow of H_2_S, the orthorhombic BaAl_2_S_4_ phase can be obtained in the temperature range from 650 °C to 800 °C [89]. In BaAl_2_S_4_:Eu thin films prepared by a BaS:Eu|Al_2_S_3_ multi-layered deposition, the formation temperature of the orthorhombic phase is lowered by about 100 °C, probably due to the more intimate mixing compared to powder mixtures [40,89]. Stiles and Kamkar evaluated the performance of both phases in EL devices, and concluded that thin films consisting primarily of the cubic phase showed a higher light output, with a maximum for the films with an almost equal amount of the cubic and orthorhombic phases [102]. A clear explanation as to whether this was related to the intrinsic efficiency of both phases could not be provided. Other effects such as increased light outcoupling could also have played a role [102].

An interesting research topic is the role of oxygen in BaAl_2_S_4_:Eu thin films. In the early days, a significant fraction of oxygen was unintentionally incorporated in the thin films [105], which could accumulate during annealing at the interface with the ZnS buffer layers. It was reported that the oxygen contamination at least partially originated from the Al_2_S_3_ evaporation [40] and the reactivity of Al_2_S_3_. Furthermore, interaction with other (oxygen-containing) layers in the thin film structure and with the substrate was suggested [106]. Shifting to other deposition techniques, such as sputtering from a BaS:Eu-Al target, allowed a better control of the oxygen content. Surprisingly, the stability of BaAl_2_S_4_:Eu layers was improved upon post-deposition annealing in an oxygen atmosphere [102,107], which was related to reduction of unsaturated bonds in the as-deposited devices or to the formation of a protective oxide layer [107].

#### 4.3.2. TDEL and CBB

Two main (technological) improvements, in parallel to the development of the BaAl_2_S_4_:Eu phosphor itself, allowed a better reproducibility and enhanced performance considerably, namely the use of thick dielectrics (TDEL) and the color-by-blue (CBB) pixel scheme.

The original EL structures, as used in the 20th century, consisted of thin film insulator layers with a thickness of only a few hundred nanometers. Two main disadvantages are associated to this concept [108]: the thin films are prone to destructive dielectric breakdown due to the high electric fields involved and should therefore be pinhole and defect free. Secondly, the use of a plane parallel thin film structure results in – mostly unwanted – optical interference effects. This leads to changes of the emission spectrum with viewing angle and with time and a dependence of the spectrum on the exact thickness of the different films [109]. Even more severely, a large fraction of the light is trapped inside the thin film structure by total internal reflections and most of the light is emitted laterally [110].

The development of a TDEL structure, in which the thin film insulator is replaced by a thick (~10-20 µm) dielectric, allowed operation of the device at higher voltages, improved the temperature resistance and significantly increased the light output due to diffuse outcoupling [108]. The advantages of thick dielectrics had already been shown in the early 1990s by Minami *et al.*, where the use of BaTiO_3_ ceramic sheets allowed high annealing temperatures, required to crystallize oxide phosphors [111,112]. Furthermore, a thick dielectric insulator, which is more tolerant towards defects, could be deposited with cheap and easily scalable screen-printing techniques [113]. More details on contrast enhancement (to counteract the reduced contrast due to the increased diffuse outcoupling) can be found in the review of (TD)EL technology by Heikenfeld and Steckl [108].

**Figure 4:**
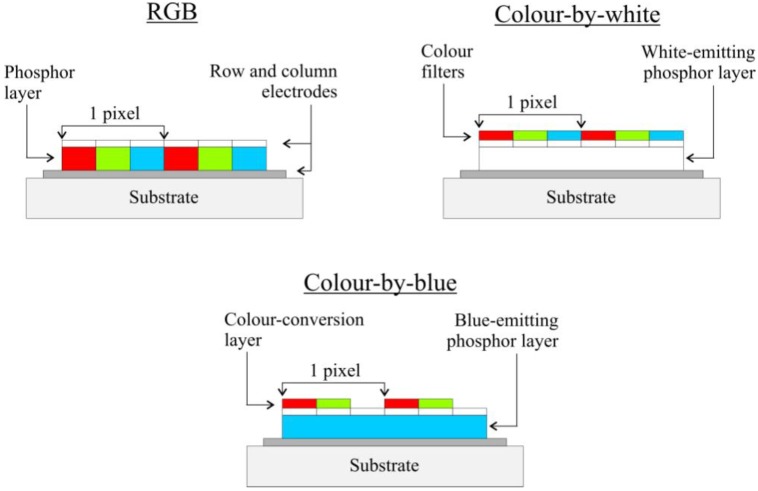
Pixel layout for thin film electroluminescence displays, with RGB subpixels **(upper left)**, colour-by-white **(upper right)** and by using a color-by-blue approach **(bottom)**. (color online)

The performance of BaAl_2_S_4_:Eu as a blue phosphor for EL, in combination with the TDEL approach, turned out to be so good that a new device structure, based on only one emitting material, could be introduced. Instead of using two or three different electroluminescent phosphor materials for the production of full-color EL displays, a color-by-blue (CBB) approach was developed [102,113]. In this way all three (RGB) sub-pixels are based on the EL emission in BaAl_2_S_4_:Eu, with photoluminescent layers (outside the electrically active structure) converting the blue emission to red and green (Figure 4). This down-conversion concept was already shown in the 1990’s by using an UV-emitting EL phosphor (ZnF_2_:Gd) in combination with one or more photoluminescent materials [114,115,116]. The CBB concept eliminates the effects of color shifts caused by differential ageing of different EL phosphor materials during the lifetime of the device. Furthermore, no subsequent patterning and deposition of the phosphor layers is required [102]. On top of the non-converted subpixels, a color correcting filter can be deposited to improve the color saturation [108]. To reduce the color blur, caused by the excitation of the conversion material by light from neighboring blue subpixels, screen-printing black stripes in between the subpixels was proposed [117]. It is interesting to compare the CBB approach to earlier attempts to use a color-by-white approach (Figure 4) [50]. In this case, a single white phosphor [118] or a stack of multi-color phosphors [119] is used to produce white light emission for every sub-pixel. Then color filters are used to filter out saturated R, G and B colors. This has the advantage, compared to an RGB-phosphor approach, that no consecutive etching and deposition of the phosphor layer is required. A disadvantage is a relatively large loss in efficiency by filtering, which occurs for all sub-pixels. In CBB, the advantage of a single emissive material for all sub-pixels is combined with high efficiency, apart from the (Stokes) conversion losses in the G and R sub-pixels.

The state-of-the art in inorganic electroluminescence displays was recently described by Hamada *et al.* [117,120]. The sputtered blue BaAl_2_S_4_:Eu phosphor layer shows a high luminance and efficiency of 2300 cd/m² and 2.5 lum/W respectively, when measured at 120 Hz and 60 V above threshold. After applying color conversion materials and a color filter, a full-color device with a peak luminance of 350 cd/m² (400 cd/m²) could be obtained for an NTSC color gamut of 100% (95%), in combination with a wide viewing angle.

#### 4.3.3. Current research activities.

(Academic) research has diminished in recent years in the field of inorganic electroluminescence in general, but also on the BaAl_2_S_4_:Eu-based phosphor in thin film form. However, several groups have worked on BaAl_2_S_4_:Eu powders. As these powders cannot be used as source material for the deposition of thin films, it merely serves to improve knowledge about the material itself.

Although BaAl_2_S_4_ powder can be prepared from a mixture of BaS and Al_2_S_3_ under a flowing H_2_S atmosphere [89], the undesired formation of Al_2_O_3_ should be suppressed by using vacuum sealed silica tubes [121]. The orthorhombic or cubic phase can be obtained by variation of the synthesis temperature [89,121]. Several other synthesis techniques were proposed, such as using Al instead of the hygroscopic Al_2_S_3_ [55]. During the synthesis, the Al precursor liquefies and lowers the synthesis temperature of the cubic BaAl_2_S_4_ phase to 660 °C [122]. Adding a H_3_BO_3_ flux, this formation temperature can be further lowered to 600 °C [122]. Other methods for the synthesis of BaAl_2_S_4_:Eu rely on a solution based approach for the synthesis of the BaS:Eu precursor [123], or on a sulfurization in a CS_2_ atmosphere of a Ba-Al-Eu oxide precursor prepared by a polymerizable complex method [124].

The radiative properties of (cubic) BaAl_2_S_4_:Eu powder were studied in detail by Barthou *et al.* [55], regarding the 5d energy level structure and the temperature dependency of the decay and the shape of the emission spectrum (via the phonon energy). The emission spectrum and decay profile for the cubic and the orthorhombic phase are very similar [89,102]. Main differences can be noticed in the excitation spectrum and a small variation in the optical band gap [89,125].

It is interesting to note that the thermal quenching of the cubic phase is still relatively limited at 500 K (*i.e.* the emission intensity has dropped by 35% compared to the low temperature intensity [55]). Taking this into account, its use as LED conversion phosphor was highlighted [126]. Nevertheless, it appears that better alternatives for the difficult to synthesize and unstable BaAl_2_S_4_:Eu powder are already available, as obtaining blue emission from Eu^2+^ is relatively common in stable, oxide hosts [127].

### 4.4. Other hosts and approaches

Several other thin film electroluminescent materials were proposed in the past decade. Ba_2_SiS_4_:Ce shows a deep blue emission, but the luminance is low [128]. Furthermore, the emission efficiency and solubility of Ce^3+^ in thiosilicate materials appears much less than that of Eu^2+^ [129], although Al^3+^ codoping might be beneficial for the incorporation of elevated concentrations of Ce^3+^ [130].

CaAl_2_S_4_:Eu,Gd was reported as an efficient green TFEL phosphor, with a luminance of 3000 cd/m² at 1 kHz and was prepared with a dual source e-beam technique [131]. With this phosphor, a wider color gamut can be obtained in comparison to SrGa_2_S_4_:Eu [132]. SrY_2_S_4_:Eu, Ca(In,Al)_2_S_4_:Eu and CuAlS_2_:Mn were investigated as red phosphor [132].

In spite of considerable advances in the deposition techniques for BaAl_2_S_4_:Eu thin films, a relatively high temperature step is still required to obtain sufficiently crystalline materials, either during deposition or during annealing. Hence, flexible substrates cannot be used under these conditions. If flexible, inorganic EL displays could be realized, this would give the technology a unique selling point over LCD and plasma displays. A sphere supported TFEL approach was proposed to obtain flexible displays, based on the deposition of the EL active layer on small dielectric BaTiO_3_ spheres (at elevated temperature), which are then transferred onto a flexible substrate and electrically contacted [133].

### 4.5. Future of iEL.

After several decades of iEL research, a good blue phosphor with reasonable efficiency is finally available. In combination with an improved (TDEL) device structure and contrast enhancement, iEL displays as presented by iFire are now state-of-the-art [117]. In 2003, Heikenfeld and Steckl labeled the iEL displays as being ‘at the crossroads’, where they would either remain a niche application or finally go for large-scale commercialization and wide market penetration [108]. Seven years later, one has to conclude that iEL did not follow the second road. LCDs have conquered the market of large displays (>30’’), initially targeted by iFire with its 34’’ pilot plant [113]. They have combined an almost continuous dropping consumer price with an increasing performance. Power consumption is reduced and contrast increased by the emerging LED backlight technology.

Given that the cost of an iEL display is for a large fraction determined by the temperature demands for the substrate and the expensive electronic circuitry, there are no prospects for (near) future market penetration, certainly because iEL still has to be considered as an invasive technology [108]. Niche applications, where the full potential of iEL devices is appreciated (such as wide temperature operating range, ruggedness and long lifetime) remain of course possible.

## 5. Color Conversion Phosphors

As described in the previous Section, several sulfide materials have been intensively investigated as thin film electroluminescent phosphors. Recently, the search for efficient color conversion phosphors for white light emitting diodes (w-LEDs) has sparked renewed interest in the photoluminescence behavior of (mainly rare-earth) doped sulfides.

w-LEDs are expected to replace incandescent light bulbs and even fluorescent lamps on a relatively short time scale. First of all, w-LEDs have many advantages, such as a high efficiency (and thus low energy consumption), small size, long lifetime (50.000h) and the absence of mercury. Incandescent light bulbs have a luminous efficacy of only 10-15 lumen per watt of electrical input power, while compact fluorescent lamps reach 40-50 lum/W. Currently, LEDs with efficiencies of over 100 lum/W have been reported, and the theoretical limit seems to be situated well above 200 lum/W, provided suitable phosphor materials can be developed. As a consequence, huge power savings (and associated reductions in fossil fuel consumption and carbon dioxide emissions) can be obtained [134].

### 5.1. Requirements for LED phosphors.

**Figure 5:**
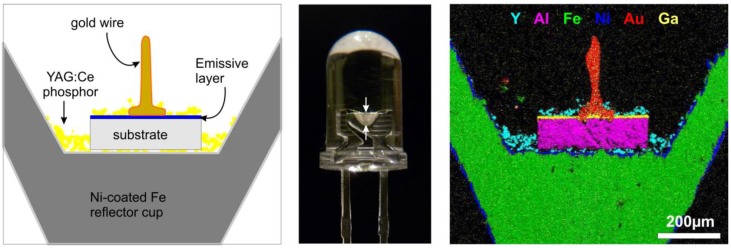
**(center)** 5mm white LED, **(left)** Schematic structure of the LED’s cross-section along the plane perpendicular to the (center) image and indicated by the white arrows, **(right)** Elemental mapping of selected elements using EDX (energy-dispersive x-ray analysis), with the maps for Y, Al and Ga indicating the Y_3_Al_5_O_12_:Ce phosphor powder, the sapphire substrate and the (Ga,In)N diode, respectively.

wLEDs are composed of a near-UV (or blue) LED, in combination with one or more phosphor materials which fully (or partially) convert the LED emission to longer wavelengths (Figure 5). An appropriate choice of the phosphor composition then results in white light emission, ideally with a high color rendering and the desired color temperature [135].

Currently, most wLEDs are based on yellow-emitting Y_3_Al_5_O_12_:Ce^3+^ (YAG:Ce) as color conversion material. Although its emission is relatively broad, it lacks a significant output in the long wavelength range of the visible spectrum, thus hampering the development of wLEDs with high color rendering and/or low color temperature. The main requirements for a color conversion material are:

1. An appropriate emission spectrum to achieve a true white emission when mixed with the remaining (visible) LED emission and possible other phosphors. To achieve a high color rendering index (CRI) for high-quality illumination applications (typically 90 or higher), broad band emission is required.
2. High quantum efficiency for the conversion process. YAG:Ce can be considered as a benchmark, with a quantum efficiency exceeding 90% [136].
3. The excitation spectrum should show sufficient overlap with the LED’s emission spectrum. As the LED’s emission spectrum can significantly change as a function of temperature and/or driving current, a broad excitation band overlapping the LED’s emission is preferred to avoid color shifts of the LED-phosphor combination.
4. A relatively short decay time, to prevent saturation in high flux devices.
5. A high thermal quenching temperature, as LED chips can reach relatively high temperatures of 450 K [137] during operation.
6. Good stability during the full lifetime of an LED (typically over 50,000 hours)

The overall efficiency for a wLED is mainly determined by the electrical-to-optical conversion efficiency of the pumping LED, the Stokes losses associated with the color conversion, the light extraction efficiency and the quantum efficiency of the phosphor. As the overall efficiency is the product of all partial efficiencies, it is of utmost importance to carefully select and optimize phosphors for a quantum efficiency as close to unity as possible, also at elevated operating temperatures.

Requirements 1, 3 and 4 favor the broad band emitting rare earth ions Ce^3+^ and Eu^2+^ over most line-emitting rare earth ions (including Eu^3+^), Mn^2+^ and transition elements. The decay times of Ce^3+^ (typically 60ns or less, [138]) and Eu^2+^ (typically 1µs or less, [139]) are sufficiently short to avoid saturation. Furthermore, these ions present a relatively small Stokes shift, which allows pumping by a blue LED which reduces conversion losses over UV LEDs, even if the quantum efficiency is close to unity. Nevertheless, the excitation band width in most compounds is sufficiently broad to allow near-UV excitation as well.

As there are several sulfide materials which can yield orange-to-red emission, these materials were recently investigated as conversion phosphor. This ability stands in contrast to oxide hosts, where red Eu^2+^ emission is relatively rare [127]. In general, the main criteria for the evaluation of rare-earth doped sulfides are requirements 5 and 6. Depending on the host’s composition, the band gap in sulfides is relatively low, typically in the range from 3 to 5eV. This implies an increased chance of interaction of the Eu^2+^ 5d orbitals with the conduction band states, thus leading to anomalous emission [140] or a relatively low quenching temperature. Furthermore, the stability of sulfides is often a matter of concern as well. In the following discussion, several host materials are discussed in the framework of the above mentioned requirements. This Section concludes with a comparison to other host compositions, such as the nitride and oxynitrides.

### 5.2. Binary sulfides

The luminescence of impurity doped binary, alkaline earth sulfides like MgS, CaS, SrS and BaS has been extensively studied in the past century. For instance, the rare earths ions (broad band d-f emitters like Eu^2+^ and Ce^3+^, as well as narrow line f-f emitters), transition metals (Cu^+^, Ag^+^, Mn^2+^, Au^+^, Cd^2+^) and s² ions (Bi^3+^, Pb^2+^, Sb^3+^,Sn^2+^) are all known to luminesce in one or more of the above mentioned hosts [141]. Several of these host-dopant combinations were studied as thin film EL phosphor (Table 1). Interestingly, undoped CaS and SrS are reported to luminesce as well, although the intensity is far too low for (LED) applications. The emission wavelength strongly depends on the synthesis conditions, suggesting the presence of multiple, optically active centers [142,143].

The emission properties of the abovementioned transition metals (except Mn^2+^) and the s^2^ ions are not very useful for LED color conversion purposes, as they often show considerable thermal quenching, in combination with a strong temperature dependent spectrum. The latter effect is due to formation of different emission centers, depending on the dopant concentration and the synthesis conditions [76,144].

In Table 2, luminescence of alkaline earth sulfides doped with some typical dopants is given. Especially the data on Eu^2+^ and Ce^3+^ doping are interesting as this is valuable information when considering the emission of these ions in ternary sulfides.

**Table 2:** Peak emission wavelength (nm) for Eu^2+^, Ce^3+^, Cu^+^ and Sb^3+^ doped alkaline earth sulfides, at room temperature and for low dopant concentration.

HostDopant	**Eu^2+^**		**Ce^3+^**		**Cu^+^**		**Sb^3+^**	
**MgS**	591	[145]	521	[146]	472	[147]	539	[148]
**CaS**	652 663	[145] [149,150]	509 520	[151] [146]	413	[147]	549	[148]
**SrS**	620	[145]	483 503	[151] [146]	478	[147]	600	[148]
**BaS**	878	[152]	480	[146]	585	[147]		

In general, the emission of sulfides shifts to longer wavelength when the cation is changed from Ba over Sr to Ca, as is the case for thioaluminates, thiosilicates and thiogallates (hereafter called the ternary sulfides). However, this observation only partially applies to the case of the alkaline earth sulfides, as can be observed in Table 2. The peak emission of SrS:Eu (620 nm) is indeed blue-shifted compared to CaS:Eu (655 nm) [152]. Although the emission of BaS:Eu was reported at 572 nm [146], this observation could not be confirmed later on [152,153]. BaS:Eu shows a broad emission band, peaking at about 878 nm, with a much longer lifetime than expected for Eu^2+^. Interaction between the 5d excited state of Eu^2+^ and the conduction band levels leads to anomalous emission [140,152]. The emission in MgS:Eu peaks at 615 nm, which is blue-shifted compared to CaS:Eu. As Ca_1-x_Mg_x_S:Eu indeed shows an increasing red-shift upon increasing the compositional parameter x from 0 to 0.5, it was concluded that the substitutional incorporation of the Eu^2+^ on the much smaller Mg^2+^ lattice led to a stress-related blue-shift in MgS:Eu [146]. Upon doping with Ce^3+^, the emission is bluish-green in SrS and green in CaS [154].

CaS:Eu, SrS:Eu and the solid solutions Ca_1-x_Sr_x_S:Eu have been in the picture as the red color conversion material in LEDs [150,154,155,156,157], due to their excitation and emission behavior, *i.e.* the emission spectrum of a blue LED perfectly overlaps with the excitation to the lowest 5d state and the emission is situated at longer wavelengths than YAG:Ce. Xia *et al.* recently determined the quantum efficiency of CaS:Eu at 53% under excitation centered at 460 nm [150]. The emission of CaS:Eu is saturated red (CIE (x,y) = (0.69, 0.30)), which can be useful for improving the color rendering and achieving low color temperatures. However, a low photopic luminous efficacy (PLE) of only 75 lum/W is a serious trade-off, as the eye sensitivity is low in this part of the visible spectrum [60]. The emission of SrS:Eu is blue-shifted, resulting in a luminous efficacy of 217 lum/W. This effect is partially counteracted by the lower quantum efficiency of SrS:Eu (31%) [150]. The emission color can be tuned, by making solid solutions of CaS:Eu and SrS:Eu, although the emission wavelength does not shift linearly on the composition, as was reported by several groups [60,146,150,155]. By adding Ce^3+^ to CaS:Eu or SrS:Eu, the excitation efficiency of the Eu^2+^ emission is enhanced, although it does not change the emission spectrum, due to an efficient energy transfer from Ce^3+^ to Eu^2+^ [154].

In contrast to the europium-doped ternary sulfides (where the optimum dopant concentration is typically around 5 mole %), concentration quenching is relatively strong in the alkaline earth sulfides, with optimum dopant concentration below 0.5% [61,158]. On the one hand this can be explained by the larger number of available cation sites within a distance of 0.4 to 0.5 nm in the alkaline earth sulfides, compared to the case of the ternary sulfides. On the other hand, this is somewhat surprising given that Eu is easily incorporated in CaS and SrS, as no charge compensation is required and as the ionic radii of Eu^2+^ and Sr^2+^ (and to a somewhat lesser extent Ca^2+^) are very similar. Although higher dopant concentrations are favorable for efficient absorption of the excitation light, it negatively affects the thermal quenching behavior. In thin films, clustering of Eu dopant ions has been reported [61], thus increasing the local concentration.

Xia reported a reduction of the emission intensity by about 40% at room temperature, compared to the intensity at 20 K, for a dopant concentration of 0.3 mole % in Ca_0.8_Sr_0.2_S:Eu [150]. Nevertheless, the emission was only quenched to 50% at 420 K, in comparison to the low temperature case. This observation, typical for Ca_1-x_Sr_x_S:Eu, shows that the thermal quenching profile deviates from the profile observed in most ternary compounds where the thermal quenching manifests itself in a rather narrow temperature region. Given that the T_0.5_ (*i.e.* the temperature when the emission intensity is halved compared to the low temperature case) has been reported at 475 K for CaS:Eu and 320 K for SrS:Eu [159], it is questionable whether Ca_1-x_Sr_x_S:Eu phosphors with higher Sr concentrations are ideal for use in LEDs.

The stability of the alkaline earth sulfides CaS and SrS is reasonable, although slow decomposition in moist air is observed. In the case of MgS:Eu, Kasano *et al.* reported its stability to be much better when the powder was fully sulfurized [146]. Besides the reduction in light output, Shin *et al.* reported another detrimental effect of the decomposition of CaS:Eu upon application in LEDs, namely a chemical reaction of the released H_2_S with the Ag pad under the LED chip, thus reducing the reflectivity [160]. Several encapsulation methods have been recently proposed, all reducing the decomposition rate under accelerated ageing conditions (high temperature and high humidity). These methods include coating with Al_2_O_3_ using atomic layer deposition [161], an organic-SiO_2_ nanocomposite [162,163] or a thin BN sheet [164].

### 5.3. Thiogallates.

In the early 1970’s, the luminescence of several europium-doped thiogallates was described (Table 3), with the peak emission wavelength red-shifting when going from BaGa_2_S_4_:Eu (490 nm) over SrGa_2_S_4_:Eu (538 nm) to CaGa_2_S_4_:Eu (560 nm) [165,166]. Some luminescent thiogallate phosphors with deviating 1:2:4 stoichiometry can also be synthesized, such as Sr_2_Ga_2_S_5_:Eu and BaGa_4_S_7_:Eu, while other compositions are thermally quenched at room temperature (e.g. Ba_3_Ga_2_S_6_:Eu and Ba_4_Ga_2_S_7_:Eu) [167].

**Table 3:** Emission properties of Eu^2+^ and Ce^3+^ doped thiogallate phosphors. *x*% quenching indicates the fraction of the emission intensity at room temperature compared to the low temperature intensity. *x*K is the temperature for which the emission intensity is half that at low temperature.

Host	Dopant	λ_max_(nm)	Quenching	Remarks	Ref.
MgGa_2_S_4_	Eu^2+^	660			[168]
CaGa_2_S_4_	Eu^2+^ Ce^3+^	565 459	410 K		[91] [169]
SrGa_2_S_4_	Eu^2+^ Ce^3+^	534 445	470 K		[95] [170]
Sr_2_Ga_2_S_5_	Eu^2+^	553 (90 K)	280 K		[166]
BaGa_4_S_7_	Eu^2+^	482 (90 K)	70%		[166]
BaGa_2_S_4_	Eu^2+^ Ce^3+^	493 448	420 K		[171] [170]
Ba_2_Ga_2_S_5_	Eu^2+^	-		No emission at 90 K	[167]
Ba_3_Ga_2_S_6_	Eu^2+^	538 (90 K)	140 K		[167]
Ba_4_Ga_2_S_7_	Eu^2+^	654 (90 K)	110 K		[167]
Ba_5_Ga_2_S_8_	Eu^2+^	-		No emission at 90 K	[167]
EuGa_2_S_4_	Eu^2+^	546	+/- 150 K		[166,172]
ZnGa_2_S_4_	Eu^2+^	540			[173]

As the quantum efficiency of CaGa_2_S_4_:Eu and Sr_2_Ga_2_S_5_:Eu was reported to be similar to that of YAG:Ce [174], the thiogallates recently attracted attention as color conversion phosphor as well [174,175,176,177,178,179]. The emission band width is considerably smaller than in YAG:Ce, which necessitates the use of at least a second, red-emitting phosphor to produce white light starting from a blue LED. Several authors reported the combination of CaS:Eu and CaGa_2_S_4_:Eu [175,177,179], even with a ‘one pot synthesis’ based on the observation that a CaS:Ga_2_S_3_ starting ratio higher than 1:1 leads to a mixture of CaS:Eu and CaGa_2_S_4_:Eu. Taking into account that CaS:Eu has a much more severe concentration quenching behavior compared to CaGa_2_S_4_:Eu, it is questionable whether this synthesis approach is optimal in terms of overall quantum efficiency of the mixture.

Yu *et al.* described the structural and luminescent properties of Ca_1-x_Sr_x_(Ga_1-y_Al_y_)_2_S_4_:Eu^2+^ phosphors [174]. Changing the values of x and y, a single crystallographic phase is obtained over the entire range. The luminescence shifts almost linearly on the composition, which allows continuous tuning of the peak emission wavelength from 496 nm (SrAl_2_S_4_:Eu) to 556 nm (CaGa_2_S_4_:Eu), while keeping a narrow emission band, indicated by a FWHM of about 40 nm.

In view of LED applications, the thermal quenching behavior is reasonable for the MGa_2_S_4_:Eu compounds (T_0.5_ = 420 K for BaGa_2_S_4_:Eu [171], 470 K for SrGa_2_S_4_:Eu [95] and 400 K for CaGa_2_S_4_:Eu [180]) allowing remote phosphor approaches, while it is worse for thiogallates with different alkaline earth to gallium ratio [167].

### 5.4. Thioaluminates and thioindates.

When BaAl_2_S_4_:Eu came in the picture as thin film electroluminescent material (Section 4), it also sparked interest in the other thioaluminates, which finally led to investigations as color conversion phosphor [126]. The pure green emission from CaAl_2_S_4_:Eu (peaking at 516 nm) makes it an interesting phosphor (Table 4). In powder from, it can be synthesized from CaS and Al_2_S_3_ under H_2_S, however this leads to the undesired formation of Al_2_O_3_ and unreacted CaS remains [181,182]. Starting from stable Al and S powder, in combination with the use of vacuum sealed silica tubes, Al_2_O_3_ formation can be suppressed. CaAl_2_S_4_:Eu shows a broad excitation spectrum in combination with a small Stokes shift, allowing overlap with both blue and near-UV pump LEDs. Upon doping CaAl_2_S_4_ with Ce^3+^, two spin-orbit split emission bands at 436 and 477 nm are observed. The emission of CaAl_2_S_4_:Ce^3+^ was however much weaker than CaAl_2_S_4_:Eu [182]. SrAl_2_S_4_:Eu shows bluish-green color with emission peaking at 495 nm [97,126].

**Table 4:** Emission properties of Eu^2+^ and Ce^3+^ doped thioaluminate and thioindate phosphors. *x*% quenching indicates the fraction of the emission intensity at room temperature compared to the low temperature intensity. *x*K is the temperature for which the emission intensity is half that at low temperature.

Host	Dopant	λ_max_(nm)	Quenching	Remarks	Ref.
MgAl_2_S_4_	Eu^2+^	499			[131]
CaAl_2_S_4_	Eu^2+^ Ce^3+^	516 436	93-98%		[97,181] [126]
SrAl_2_S_4_	Eu^2+^	495	90%		[97]
Sr_2_Al_2_S_5_	Eu^2+^	521			[97]
BaAl_4_S_7_	Eu^2+^	470	490 K		[97]
BaAl_2_S_4_	Eu^2+^	467-473 473	>550 K	Cubic Orthorhombic	[55,89,97] [89]
Ba_2_Al_2_S_5_	Eu^2+^	487			[97]
Ba_4_Al_2_S_7_	Eu^2+^	534	330 K		[97]
Ba_5_Al_2_S_8_	Eu^2+^	540			[97]
EuAl_2_S_4_	Eu^2+^	508			[166]
CaIn_2_S_4_	Eu^2+^	731	strong	95 nm FWHM	[183]
SrIn_2_S_4_	Eu^2+^	640 (80 K) 614	strong		[166] [183]
BaIn_2_S_4_	Eu^2+^	680 (80 K) 663	strong	145 nm FWHM	[166] [183]

The preparation requirements and the intrinsic instability of the phosphor against moisture make handling difficult and potentially reduce the lifetime upon improper encapsulation. In combination with the reported moderate quantum efficiencies [97,124,181], make the class of the thioaluminates less suited for LED color conversion, in spite of the excellent thermal quenching behavior of for instance BaAl_2_S_4_:Eu [55] (Table 4).

Upon consideration of the interesting properties of the alkaline earth thioaluminate and thiogallate phosphors, one might also consider the thioindate phosphors, for which a red-shifted emission can be anticipated. However, the relatively small bandgap of these materials compared to the thioaluminates and thiogallates [184], leads to anomalous emission and/or strong thermal quenching due to the increased interaction of the 5d excited states of Eu^2+^ and the conduction band states [159]. For instance BaIn_2_S_4_ and CaIn_2_S_4_, both show a strongly broadened and weak emission at room temperature [166,183].

### 5.5. Thiosilicates and thiogermanates.

**Figure 6:**
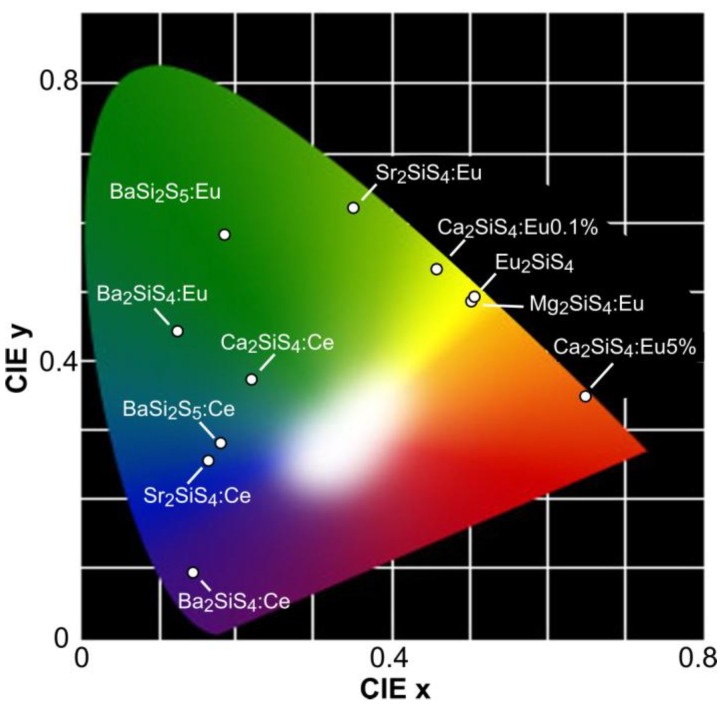
Emission colors of europium and cerium doped thiosilicates. Dopant concentration is 1mol %, unless otherwise specified [129,186,188].

The emission of thiosilicate hosts upon doping with Ce^3+^ and Eu^2+^ covers the entire range from deep-blue (Ba_2_SiS_4_:Ce^3+^) to saturated red (Ca_2_SiS_4_:Eu^2+^) [129,168]. Figure 6 gives an overview of the emission colors that can be obtained. In general, Ce^3+^ doping is less efficient and the optimal dopant concentration is fairly low. Adding Al^3+^ as a co-dopant to Ba_2_SiS_4_:Ce^3+^ improves the crystallinity, the luminescence intensity and the dopant incorporation [130]. An internal quantum efficiency of 36% was reported.

Ca_2_SiS_4_:Eu^2+^ shows two emission bands, at 565 nm and 660 nm [186], with the ratio between both bands depending on the europium concentration (Table 5). The solubility of Eu^2+^ in the orthorhombic Ca_2_SiS_4_ host is limited to about 10%. For higher concentrations, a second, monoclinic phase similar to Eu_2_SiS_4_ is formed [189]. The fully substituted compound Eu_2_SiS_4_ is still luminescent, although the emission efficiency is considerably quenched [186,190]. Moreover, the practical application of such a fully substituted phosphor would be limited due to the prohibitively high cost of europium. It is interesting to note that also EuGa_2_S_4_ and EuAl_2_S_4_ show luminescence at room temperature, while EuS does not [166,191,192].

**Table 5:** Emission properties of Eu^2+^ and Ce^3+^ doped thiosilicate and thiogermanate phosphors. *x* % quenching indicates the fraction of the emission intensity at room temperature compared to the low temperature intensity. *x*K is the temperature for which the emission intensity is half that at low temperature.

Host	Dopant	λ_max_(nm)	Quenching	Remarks	Ref.
Na_2_Si_2_S_5_	Eu^2+^	488		Weak emission	[185]
Na_4_SiS_4_	Eu^2+^	460			[185]
	Ce^3+^	436			
Mg_2_SiS_4_	Eu^2+^	660			[168]
Ca_2_SiS_4_	Eu^2+^	565	445 K	[Eu] < 1 mol %	[186,187]
	Eu^2+^	660	470 K	[Eu] > 1 mol %	[186,187]
	Ce^3+^	475			[129]
CaEuSiS_4_	Eu^2+^	614			[186]
Sr_2_SiS_4_	Eu^2+^	545	380 K		[168,188]
	Ce^3+^	466			[168]
SrSi_2_S_5_	Eu^2+^	490			[185]
Ba_2_SiS_4_	Eu^2+^	495			[129,185]
	Ce^3+^	435			[129,185]
BaSi_2_S_5_	Eu^2+^	505			[129,185]
	Ce^3+^	508			[129,185]
Ba_3_SiS_5_	Eu^2+^	-		No emission at RT	[128]
Eu_2_SiS_4_		577			[186]
Sr_2_GeS_4_	Eu^2+^	-		No emission at RT	[185]
Ba_2_GeS_4_	Eu^2+^	-		No emission at RT	[185]

The stability of the thiosilicates upon contact with moisture depends on the composition. It is significantly better than the thioaluminates, and comparable to that of CaS and SrS. Several thiosilicates show two emission bands with the ratio depending on the europium concentration, often leading to relatively broad emission. This might be favorable for use as LED phosphor, where one can tune the emission color by changing both the composition of the host lattice and the dopant concentration. Ca_2_SiS_4_ and Sr_2_SiS_4_ show only a partial miscibility, due to a different crystal lattice [188]. The thermal quenching is limited for Ca_2_SiS_4_:Eu (T_0.5_ = 470 K) [187], but appears worse for other compounds like Sr_2_SiS_4_:Eu (T_0.5_ = 380 K) [188]. At 425 K, Ba_2_SiS_4_:Ce^3+^ keeps 80% of its room temperature emission intensity [130].

Only a few reports on the luminescence of the thiogermanates are available. Ba_2_GeS_4_:Eu^2+^ and Sr_2_GeS_4_:Eu^2+^ are not luminescent at room temperature [185]. A much smaller optical band gap can be anticipated for the thiogermanates in comparison to the thiosilicates. This would lead to a strong interaction (if not overlap) between the 5d excited states and the conduction band states and consequently to a strong thermal, if not full, quenching of the luminescence.

### 5.7. Future

The past years were characterized by a strong research interest in color conversion phosphors for LEDs. The lack of phosphors with sufficient emission intensity in the red part of the visible spectrum can be overcome with certain (Eu^2+^-doped) sulfide phosphors. As discussed above, several sulfide phosphors are well suited as color conversion material, provided that their thermal quenching behavior and quantum efficiency are meticulously studied. Currently, these aspects are often lacking in recently published work on (sulfide) conversion phosphors. When compared to other recently proposed hosts, such as the nitrides [135,193,194] and oxynitrides [195,196], the sulfides have the disadvantage of a much lower stability, although this could be improved upon proper encapsulation, which can both be achieved at the level of a single phosphor particle or by incorporation in an impermeable matrix. Of course, when considering the expected lifetime of an LED being in the range of 10-50 khours, one would rather use the most stable phosphor host available.

## 6. Persistent Luminescence and Storage Phosphors

Persistent phosphors are materials which can emit light up to hours after the (optical) excitation has ceased. Since more than a decade, green-emitting SrAl_2_O_4_:Eu,Dy has replaced ZnS:Cu,Co, due to its better stability and longer afterglow [197]. Several other efficient materials were developed, especially in the short wavelength range of the visible spectrum, such as CaAl_2_O_4_:Eu,Nd (violet, [198]) and Sr_2_MgSi_2_O_7_:Eu,Dy (blue, [199]). A review of the reported persistent phosphors and the different models which have been proposed can be found in this ‘Special Issue’ [200]. Yellow and red persistent phosphors with high initial brightness are relatively scarce, which is partially due to the reduced eye sensitivity at low light intensity levels and the limited number of host materials for Eu^2+^ yielding red emission [201]. Moreover, a new standard was proposed to accurately describe persistent phosphors when light levels are below 1 cd/m² [201,202]. The development of bright red persistent phosphors would open a new range of applications, for instance in emergency signage.

To obtain persistent luminescence in the long wavelength range of the visible spectrum, one could either look at Eu^3+^ based materials or specific Eu^2+^ activated sulfides. Y_2_O_2_S:Eu^3+^ codoped with Ti and Mg is one of the few red persistent phosphors, but it cannot efficiently be excited by visible light and the afterglow is relatively short [203].

As CaS:Eu^2+^ and SrS:Eu^2+^ are able to yield red and orange emission respectively, they have attracted some attention as persistent phosphor as well. SrS:Eu^2+^ often shows some afterglow even without (intentional) co-doping [204], which is possibly related to synthesis conditions promoting sulfur deficiency. The addition of Dy^3+^ somewhat enhances the afterglow, although it remains short and relatively weak [205,206].

The addition of Cl^-^ to CaS:Eu yields an afterglow in the deep-red region with slightly red-shifted emission compared to CaS:Eu [207]. Addition of trivalent ions such as Y^3+^, Al^3+^ and Tm^3+^ to CaS:Eu gives moderate (Y, Al) to bright afterglow (Tm) [208,209], while adding Na^+^ to CaS:Eu,Tm reduces it, suggesting a significant role for the charge compensating defects.

Upon adding Sm^3+^ to CaS:Eu [210], a photo stimulable phosphor (rather than a persistent phosphor) is obtained as the trap levels introduced by Sm^3+^ are too deep to be thermally emptied. However, upon illumination of the material with infrared excitation, visible emission can be obtained (provided the powder has previously been excited by UV or visible light, x-rays…, which discriminates it from an up conversion phosphor). The exact defect structure caused by the introduction of the rare earth ions and its influence on the energy level scheme has not been fully established yet [211,212]. Furthermore, the phosphor’s behavior strongly depends on the concentrations of the Sm and Eu dopants [213]. Jia *et al.* also reported that the addition of Tm^3+^ to Ca_0.9_Sr_0.1_S:Bi^3+^ prolongs and intensifies the (blue) afterglow in this material [214]. A relatively short and weak afterglow could be obtained upon co-doping of CaGa_2_S_4_:Eu, with the best result for Ho^3+^ [101,215].

Recently, persistent luminescence in Ca_2_SiS_4_:Eu,Nd was reported, with the main emission band peaking at 660 nm [187]. Practical usage seems limited, as excitation below 360 nm is required to induce the persistent luminescence. In addition, the perceived emission intensity is relatively low, due to the eye sensitivity which drops rapidly at decreasing light levels at this long wavelength [201]. Nevertheless, it could be interesting from a fundamental point of view, as it was shown that transfer of charge carriers via the conduction band must be limited and that the Nd^3+^ codopants (and associated charge compensating defects) most probably reside in the proximity of the Eu^2+^ ions [187].

Most of the time, persistent phosphors are applied as luminous paint, for instance in emergency exit indicators. Hence they are required to have long lifetimes in often relatively harsh environments. Even with adequate encapsulation, the intrinsic instability of phosphor hosts like CaS, SrS or Ca_2_SiS_4_ appears unfavorable for persistent luminescence applications.

## 7. Luminescent Sulfide Nanoparticles

### 7.1. Undoped nanoparticles

#### 7.1.1. Introduction

Colloidal semiconductor nanocrystals or quantum dots (Qdots) offer an interesting alternative to Ce^3+^- or Eu^2+^- doped bulk sulfide materials. When their radius *R* is smaller than the bulk exciton Bohr radius *R_B_*, their electronic properties, most importantly the Qdot band gap, become size-dependent [216]. In this regime, termed the strong quantum confinement regime, the Qdot eigenstates form a discrete set instead of the quasi-continuum of states found in bulk semiconductors. To illustrate this, let us first assume that a Qdot can be regarded as an infinite spherical quantum well. The eigenenergies *E_lk_* of a particle with mass *m_0_* in this infinite well are given by:

$$E_{lk}=\frac{\hbar^{2}u_{lk}^{2}}{2m_{0}R^{2}}$$

*u_lk_* denotes the *k*-th zero of the BesselJ-function of order *l.* Clearly, every energy level *E_lk_* increase with the inverse of the square of the particle radius *R*. A more rigorous calculation of the Qdot eigenstates accounts for a shielded Coulomb attraction between the electron and hole which constitute the exciton formed after excitation. This correction scales with *R*^-1^, and we obtain following size-dependence for the Qdot band gap *E_0_* (Brus-equation, [216] *E_g_*: bulk band gap; *m_e_*: electron effective mass; *m_h_*: hole effective mass, *ε* dielectric constant):

$$E_{0}=E_{g}+\frac{\hbar^{2}\pi^{2}}{2R^{2}}(\frac{1}{m_{e}}+\frac{1}{m_{h}})-\frac{1.8e^{2}}{\varepsilon R}$$

Conveniently, the blue shift of the band gap with decreasing Qdot size allows covering a large part of the electromagnetic spectrum by a single material. Taking the cadmium salt Qdots as an example: CdS emits violet to blue light, CdSe emits from blue to red and the CdTe emission can be tuned from green to deep red [217], [218].

Most semiconductor Qdot materials can be synthesized with a high photoluminescence quantum yield, which is often accomplished by coating the Qdot core with an inorganic shell [219]. This shell, typically consisting of ZnS, has a larger band gap than the Qdot core and thus prohibits a penetration of the exciton wavefunction into the shell (type-I core-shell Qdots, both carriers localized in the Qdot core). It effectively shields the exciton from the surface; hence, possible surface states no longer lead to a quenching of the Qdot luminescence and Qdots with a high quantum yield are obtained. For instance, quantum yield values of up to 60% have been reported for CdSe/ZnS and InP/ZnS core-shell Qdots [219].

Coating procedures can also be used to further engineer the Qdot band gap. Instead of using a high band gap shell material (such as ZnS), two materials with a large bulk band offset can be combined. In this case, the staggered band alignment leads to a spatially separated exciton, where the electron for instance remains in the Qdot core while the hole is transferred to the shell (type-II core-shell Qdots). An indirect band gap is formed which may be, depending on the core size and shell thickness, narrower than the bulk band gap of both constituents. CdS/ZnSe for instance can be synthesized with an absorption onset down to 650 nm, while both bulk materials have an absorption onset of 515 nm and 460 nm, respectively [219].

The versatile band gap tuning and high photoluminescence quantum yield has lead to a diverse range of applications based on colloidal Qdots; they are currently used as luminescent biomarkers [220,221] and in light-emitting diodes [222,223,224,225] and lasers [226,227,228]. Clearly, colloidal semiconductor Qdots have a large potential, however covering the broad range of materials and applications is beyond the scope of this review. We refer to the review by Bera *et al.* [229] for a more detailed discussion. Here, we will limit ourselves to sulfide Qdots, in casu CdS and PbS, and the applications realized with these materials.

#### 7.1.2. Brief historic overview of sulfide Qdots

**Figure 7:**
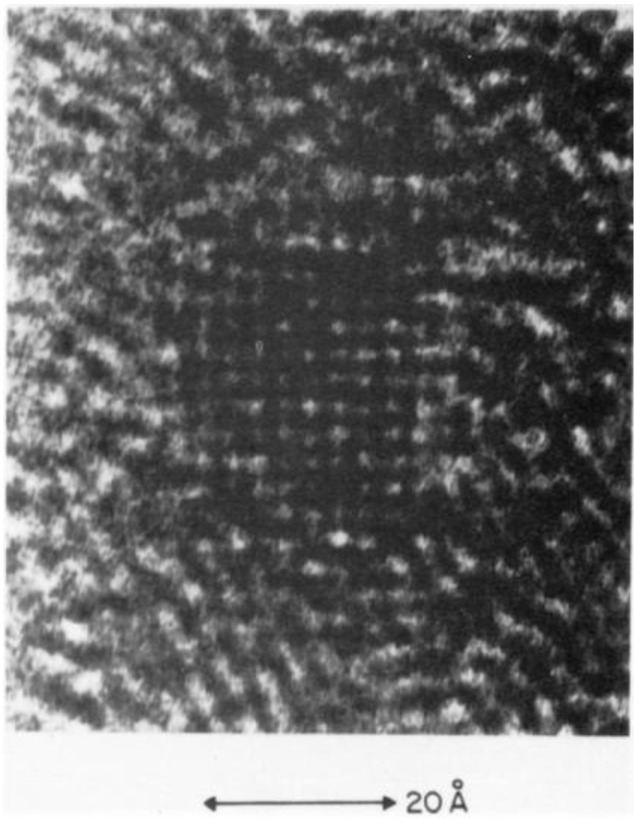
Transmission electron microscopy image of a single PbS nanocrystal (Reprinted with permission from [231]. Copyright 1985 American Institute of Physics).

**Figure 8:**
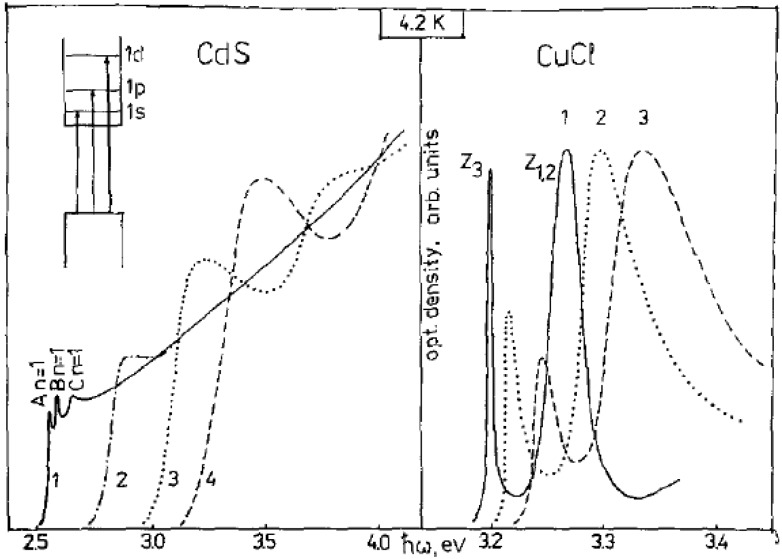
**(left)** Absorbance spectra of CdS Qdots of radius 32 nm (1), 2.3 nm (2), 1.5 nm (3) and 1.2 nm (4), **(right)** absorbance spectra of 31 nm (1), 2.9 nm (2) and 2.0 nm (3) CuCl Qdots (Reprinted from [237] with permission from Elsevier).

During the pioneering years of research on quantum dots (Qdots), now thirty years ago, metal sulfide nanocrystals were among the first materials to be synthesized. Following the groundbreaking theoretical work of Brus [216] and Efros and Efros [230], who calculated that quantum confinement induces a discretization of the electronic band structure and a blue shift of the band gap in small semiconductor nanocrystals, several research groups indeed succeeded in the synthesis of metal sulfide Qdots, more specifically CdS and PbS, either in solution [231,232,233,234], in polymers [235,236] or in a glass host [237,238,239,240]. As a result of the small nanocrystal size, typically a few nanometer (Figure 7), the Qdot band gap was clearly shifted to higher energies and pronounced absorption peaks, due to transitions between the discrete Qdot energy levels, appeared in the spectrum (Figure 8).

However, the typical Qdot photoluminescence yield in these materials was low, and the Qdots often suffered from trap emission due to the high surface-to-volume ratio. Although Henglein [232] successfully increased this yield to 50% by a coating of CdS particles with Cd(OH)_2_, this problem was remedied more efficiently by the development of a high temperature organic phase synthesis, described by Murray *et al.* in 1993 [241]. High-quality colloidal CdX (X = S, Se, Te) Qdots of uniform size were prepared by their method. Furthermore, a proper passivation of the surface by organic ligands ensured a high photoluminescence yield. Consequently, this paper sparked a strong interest in colloidal Qdots.

#### 7.1.3. State-of-the-art

**Figure 9:**
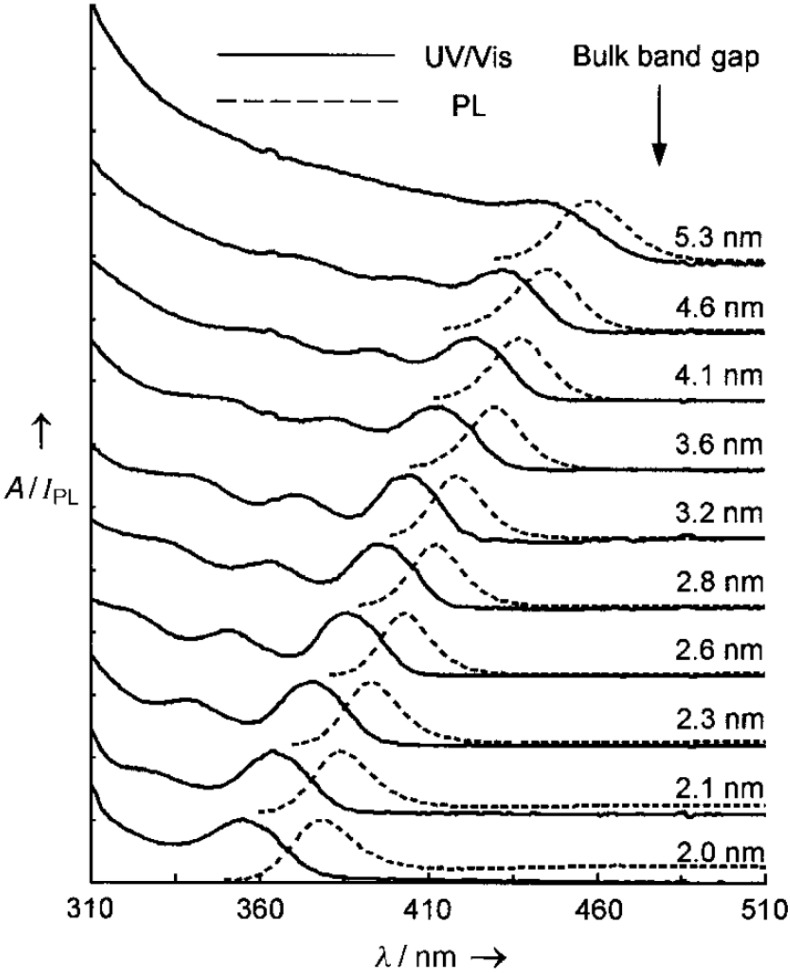
Series of absorbance (full lines) and luminescence spectra (dotted lines) of CdS Qdots of varying size. (Reprinted from [217] with permission. Copyright Wiley-VCH Verlag GmbH & Co. KGaA.).

Currently, both CdS and PbS Qdots are widely investigated, especially since the development of high quality synthesis routes employing greener chemicals. For the production of CdS Qdots, Peng *et al.* replaced the dimethyl cadmium employed by Murray *et al.* [241] with Cd-phosphonate or Cd-carboxylate complexes, which were prepared by reacting CdO with the respective long-chained acid [217,218]. As the bulk band gap for CdS equals 2.41 eV [242], these synthesis schemes usually yield Qdots with an emission tunable from the blue to the near UV (Figure 9). Reported quantum efficiencies for CdS Qdots produced by similar methods typically vary between 3 and 12% [243,244], although it can be increased to 30-40% by coating the Qdots with a protective ZnS shell [244,245]. Similarly, encapsulating them with 2-mercaptopropionic acid increases the luminescence yield to ca. 50% [246].

**Figure 10:**
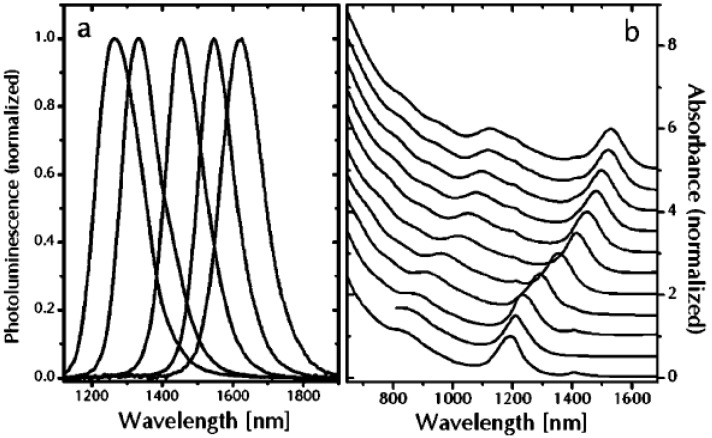
Series of luminescence (a) and absorbance spectra (b) of PbS Qdots of varying size. (Reprinted with permission from [247]. Copyright 2006 American Chemical Society).

With a bulk band gap of 0.41 eV, PbS Qdots are an ideal material for NIR imaging [248], photodetectors and photovoltaics [249,250,251,252,253] and telecom applications [227,254]. In 2003, two important colloidal synthesis routes for PbS Qdots were proposed. They differ significantly, the method of Hines *et al.* [255] being based on leadoleate and bis(trimethylsilyl)sulfide, while Joo *et al.* [256] (and later Cademartiri *et al.* [247]) start from PbCl_2_ and elemental sulphur dissolved in oleylamine. However, both lead to monodisperse PbS Qdot suspensions, indicated by the sharp absorption and luminescence features of the Qdots (Figure 10). They possess a strong band gap luminescence in the NIR (Figure 10), with quantum yields ranging between 20 and 80% [247,255,257]. Interestingly, the luminescence decay time in these materials is quite long (typically a few µs) [258], comparable to PbSe Qdots [259], but much longer than CdX materials, which have a decay time of the order of 10-50 ns [246,260,261]. Recent results have shown that a strong local field effect in PbS Qdots [262] leads to this long decay time, although the different band structure may also play a role here.

#### 7.1.4. Applications of luminescent metal sulfide Qdots

**Figure 11:**
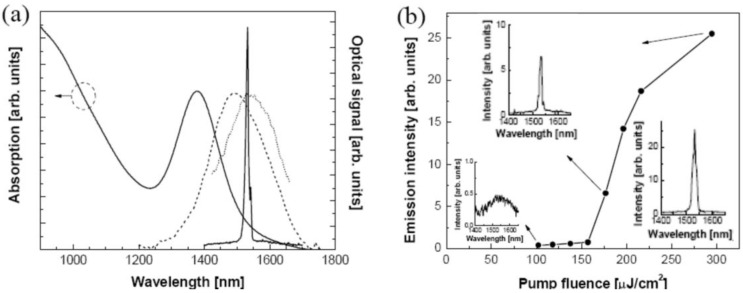
**(a)** Absorption spectrum (full line) and below-threshold photoluminescence spectrum at room temperature (dashed line) and 80 K (dotted line) of PbS Qdots. The sharp peak corresponds to the emission spectrum of the Qdot microcapillary laser when pumped above threshold. **(b)** Pump fluence dependence of the Qdot emission. The threshold behavior and representative emission spectra (insets) clearly demonstrate lasing. (permission requested, adapted from [227]).

Due to their bright luminescence, both CdS and PbS Qdots are interesting materials for photonic devices based on light emission. Hence, the observation of amplified spontaneous emission and gain in visible [260,263,264] and NIR [265,266,267] Qdot materials rapidly led to the development of Qdot lasers. For instance, using CdS Qdots, an optically pumped blue laser was recently demonstrated [228]. The Qdot emission was coupled to the whispering gallery modes of a glass microsphere, and above a threshold of ~60µW (corresponding to a fluence of 3.7 mJ/cm^2^), lasing was observed. Similarly, PbS Qdots have been used by Hoogland *et al.* [227] to produce a laser operating at telecom wavelengths (1.53 µm in this case). By coating the inner walls of a glass microcapillary tube, the emission was again coupled to the whispering gallery modes of the cavity, leading to efficient lasing above a threshold fluence of 177 µJ/cm^2^ (Figure 11).

**Figure 12:**
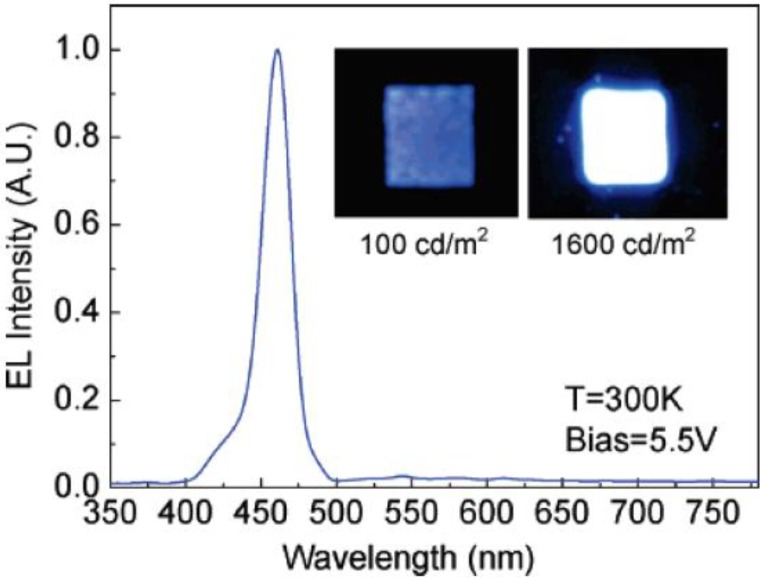
Electroluminescence spectrum of a CdS Qdot LED, measured at a bias of 5.5 V. Inset: images of the LED. (Reprinted with permission from [222]. Copyright 2007 American Chemical Society.).

Next to emission due to photoexcitation, colloidal Qdots can also be excited by electrical pumping. The demonstration of electroluminescence [268,269,270] was therefore an important step forward in the fabrication of light-emitting devices (LEDs). Following these early experiments, Qdot LEDs based on CdS (Figure 12) [222] and PbS [223] Qdots have been reported. While PbS Qdots offer the unique possibility of having efficient NIR LEDs over a wide spectral range, unfortunately, in the case of CdS, we are restricted to the blue part of the electromagnetic spectrum. Other CdX materials and compositions are needed to enable LED fabrication over the remaining visible spectrum [224,225]. However, as reported by Anikeeva *et al.*, in the blue spectral region important challenges remain, as the external quantum efficiency is low [224]. A value of only 0.4% was obtained for a blue LED employing ZnCdS Qdots coated with a ZnS shell, compared to an efficiency of 2.6% for a green LED. This implies that there is still plenty of room for optimization, either through device fabrication or an improvement of the active material.

### 7.2. Doped nanoparticles

During the past decade, the strong research effort in semiconductor quantum dots also sparked interest into doped quantum dots, being the nano-sized counterpart of the well-studied rare earth and transition metal doped bulk phosphors. Regardless of the exact composition of these nano-materials, it is not a priori clear whether reducing the particle’s size is favorable for the luminescence properties of the dopants (having localized transitions). The increase in band gap which follows the decrease in size might be favorable for the thermal quenching behavior, while the increased surface-to-volume ratio offers additional non-radiative decay routes. The main obstacle, however, is the effective incorporation of the dopant ions into the nanoparticles, rather than simply decorating the particles’ surface. As hardly any work has been reported on ternary sulfides, except for some top-down approaches, where particle size is reduced by ball-milling [271], we will focus on doped ‘simple sulfides’ like ZnS, CaS and SrS.

ZnS:Mn is probably the most studied doped sulfide in nano-sized form, due to the chemical similarity between Zn^2+^ and Mn^2+^, facilitating the incorporation of the dopant ion. Nevertheless, the incorporated concentration is consistently lower than the intended dopant concentration [272] and part of the Mn^2+^ ions reside near the surface of the nanoparticle. The work by Bol *et al.* showed that the lifetime of Mn^2+^ in ZnS:Mn^2+^ quantum dots is very similar to the bulk lifetime [273,274], in spite of earlier work stating a considerable shortening of the lifetime [275]. Also the emission spectrum itself is almost size-independent [272,274]. Hence, growing an inorganic (ZnS) shell around ZnS:Mn effectively reduces non-radiative decay paths and enhances the luminescence [276], more efficiently than organic passivation of the surface [272]. Synthesis techniques, luminescence properties and possible applications of ZnS:Mn nanoparticles can be found in the Reviews by Chen *et al.* [277] and Yang *et al.* [272].

For the solvothermal synthesis of ZnS, Biswas and Kar investigated the influence of precursors, solvents and temperature on the crystallographic phase formation (cubic and hexagonal), morphology (particles, rods and sheets) and size (few nm to micron size) [278]. Some reports are available on the luminescence of nano-sized ZnS:Eu, although the results are diverging, with the presence of both Eu^3+^ and (assumed) Eu^2+^ emission [279,280,281]. Bulk ZnS:Eu is not luminescent and it was supposed that increasing the band gap of the host would allow the 5d-4f transition in Eu^2+^. Incorporation of Eu in the first place appears difficult in ZnS, due to the strongly different ionic radius of Zn^2+^ and Eu^2+^ [282].

AC electroluminescence was reported using ZnSe/ZnS:Mn/ZnS core/shell nanoparticles with a high photoluminescent quantum efficiency of 65% [283]. The semiconductor part of the ACTFEL devices consisted of a multi-layer of spin-casted nanoparticles layers (thickness of 30 nm) and sputtered (undoped) ZnS layers (12 nm). The typical orange ZnS:Mn emission was obtained for these structures, with a brightness of 2 cd/m² at 30 kHz, while devices without the sputtered ZnS layers didn’t show EL emission. Toyama *et al.* did obtain EL in a 150 nm thick printed layer of ZnS:Mn nanoparticles. At 5 kHz, and 45 V above threshold, a luminance of 1cd/m^2^ could be obtained for the smallest particles (3 nm) [284].

The doped ‘simple sulfides’, like CaS and SrS, have recently attracted some attention in their nano-sized form. Several synthesis methods have been proposed for the synthesis of CaS and SrS, such as a precipitation method (using chloride precursors and sodium sulfide [285,286]) and an alkoxide method (using H_2_S as sulfur source [285]). With both methods, particle sizes in the range from 15 to 25 nm could be obtained. Upon doping with Eu^2+^, the as-prepared particles are not luminescent and EPR (electron-paramagnetic resonance) shows the europium ions are not incorporated in the particles yet (at least not in their divalent state). After a thermal annealing at typically 700 °C, luminescent nanoparticles are obtained, with emission and excitation spectra rather similar to the bulk counterparts [285,286]. Other methods using a reducing atmosphere at high temperature were proposed for the preparation of SrS:Eu,Dy and SrS:Cu nanoparticles [205,287].

A solvothermal method was also reported to obtain doped sulfides, such as CaS:Pb, CaS:Ag and CaS:Bi [288,289]. Chloride precursors, elemental sulfur, ethylenediamine and capping agents are heated to about 170 to 220 °C in a teflon-lined autoclave for several hours. Particles with a relatively broad size range are obtained, ranging from tens to hundreds of nm. No thermal annealing is required to obtain luminescent particles. Modifications to this solvothermal synthesis using ethylenediamine as mentioned above, allowed the formation of nicely faceted, sub-micron to micron-sized Ca_1-x_Sr_x_S single crystals [155] [149,155]. Incorporation of dopant ions (Eu^2+^ and Ce^3+^) is straightforward and leads to bright luminescence, without the need for a high-temperature annealing step. Hence, the temperature of the entire synthesis process is limited to 200 °C, and no H_2_S gas is used.

Whispering gallery modes (WGMs) were observed in Eu^2+^ and Ce^3+^-doped CaS and SrS particles, which were related to resonance modes in the equatorial planes of the octahedron shaped crystals [290]. A modification of this solvothermal method allowed the growth of oriented and textured CaS:Eu and SrS:Eu thin films, which showed bright photoluminescence, with emission properties in line with bulk phosphors, and AC electroluminescence when electrically contacted [291].

Recently, CaS:Eu nanowires with a high aspect ratio and diameter between 50 and 150 nm were prepared using a (molten) B_2_O_3_ matrix [164]. Unlike most of the nano-sized doped sulfides mentioned in this section, the Eu^2+^ emission spectrum deviates from the bulk phosphor, with a blue-shift of about 30 nm.

## 8. Conclusions

In this Review, we attempted to give an account of the rich history of sulfides for a wide range of luminescence applications. Without any doubt, the sulfides possess specific properties which made them especially suited as powder electroluminescent phosphor (based on ZnS:Cu) or as thin film electroluminescent material, where one should highlight the yellow-orange-emitting ZnS:Mn and the blue BaAl_2_S_4_:Eu phosphor. However, the future looks rather dim for these lighting and display techniques, due to the advent of superior techniques, like organic LEDs and liquid crystal displays. It is interesting to note that both in powder and thin film EL, the amount of research and development has always been relatively small, compared to the effort put into other techniques. Nevertheless, as thin film electroluminescence has some intrinsic advantages, it might still have a future as a full-color display technique in demanding environments.

The future of sulfide phosphors could be situated in the field of color conversion for white LEDs. Upon doping with Eu^2+^ and Ce^3+^, the emission can be tuned from deep blue to saturated red by appropriately choosing the host composition. In general, the emission and excitation bands are sufficiently broad, allowing both a good color rendering and efficient pumping by the LED. Two major criteria should however be systematically evaluated, namely the quantum efficiency of photoluminescence and the thermal quenching behavior (both in terms of intensity reduction and spectral shift). Even if ‘ideal’ host-dopant combinations are found, the preparation conditions and the long term stability of sulfides will largely determine whether they are to be incorporated in LEDs after all. Fine tuning of synthesis conditions and the application of protective coatings are two major research tracks to be considered.

CdS and PbS quantum dots have already shown unique abilities and future potential as luminescent material for a wide range of photonic applications, both in the visible and the near-infrared. However, from environmental point of view, the toxicity of both Pb and Cd remains a serious issue, hampering their large scale application. Doped semiconductor nanoparticles (based on sulfides) have probably not shown their full potential yet, as the incorporation of the dopants into the nanoparticles appears to be non-trivial.
